# Supplementary material for: Abnormal Dynamic Reconfiguration of Multilayer Temporal Networks in Patients with Bipolar Disorder
Source: Brain Sci. 2024 Sep 19;14(9):935. doi: 10.3390/brainsci14090935 (PMC11430687; doi:10.3390/brainsci14090935)
Supplement: Supplementary file 1 [file brainsci-14-00935-s001.zip › brainsci-3187652-supplementary.pdf]

Supplementary Materials for "Abnormal Dynamic Reconfiguration of Multilayer Temporal Networks in Patients with Bipolar Disorder"

Table S1. Brain regions and the mapping information on functional subnetworks

Labels	Regions	Abbreviation	Subnetwork
1	Precental gyrus	PreCG.L	MAN
2	Precental gyrus	PreCG.R	MAN
3	Superior frontal gyrus, dorsolateral	SFGdor.L	DMN
4	Superior frontal gyrus, dorsolateral	SFGdor.R	DMN
5	Superior frontal gyrus, orbital part	ORBsup.L	AN
6	Superior frontal gyrus, orbital part	ORBsup.R	DMN
7	Middle frontal gyrus	MFG.L	AN
8	Middle frontal gyrus	MFG.R	AN
9	Middle frontal gyrus, orbital part	ORBmid.L	AN
10	Middle frontal gyrus, orbital part	ORBmid.R	AN
11	Inferior frontal gyrus, opercular part	IFGoperc.L	AN
12	Inferior frontal gyrus, opercular part	IFGoperc.R	AN
13	Inferior frontal gyrus, triangular part	IFGtriang.L	AN
14	Inferior frontal gyrus, triangular part	IFGtriang.R	AN
15	Inferior frontal gyrus, orbital part	ORBinf.L	AN
16	Inferior frontal gyrus, orbital part	ORBinf.R	AN
17	Rolandic operculum	ROL.L	MAN
18	Rolandic operculum	ROL.R	MAN
19	Supplementary motor area	SMA.L	AN
20	Supplementary motor area	SMA.R	MAN
21	Olfactory cortex	OLF.L	LSN
22	Olfactory cortex	OLF.R	LSN
23	Superior frontal gyrus, medial	SFGmed.L	DMN
24	Superior frontal gyrus, medial	SFGmed.R	DMN
25	Superior frontal gyrus, medial orbital	ORBsupmed.L	DMN
26	Superior frontal gyrus, medial orbital	ORBsupmed.R	DMN
27	Gyrus rectus	REC.L	DMN
28	Gyrus rectus	REC.R	DMN
29	Insula	INS.L	MAN
30	Insula	INS.R	MAN
31	Anterior cingulate and paracingulate gyri	ACG.L	DMN
32	Anterior cingulate and paracingulate gyri	ACG.R	DMN
33	Median cingulate and paracingulate gyri	DCG.L	LSN
34	Median cingulate and paracingulate gyri	DCG.R	LSN

35	Posterior cingulate gyrus	PCG.L	DMN
36	Posterior cingulate gyrus	PCG.R	DMN
37	Hippocampus	HIP.L	LSN
38	Hippocampus	HIP.R	LSN
39	Parahippocampal gyrus	PHG.L	LSN
40	Parahippocampal gyrus	PHG.R	LSN
41	Amygdala	AMYG.L	LSN
42	Amygdala	AMYG.R	LSN
43	Calcarine fissure and surrounding cortex	CAL.L	VN
44	Calcarine fissure and surrounding cortex	CAL.R	VN
45	Cuneus	CUN.L	VN
46	Cuneus	CUN.R	VN
47	Lingual gyrus	LING.L	VN
48	Lingual gyrus	LING.R	VN
49	Superior occipital gyrus	SOG.L	VN
50	Superior occipital gyrus	SOG.R	VN
51	Middle occipital gyrus	MOG.L	VN
52	Middle occipital gyrus	MOG.R	VN
53	Inferior occipital gyrus	IOG.L	VN
54	Inferior occipital gyrus	IOG.R	VN
55	Fusiform gyrus	FFG.L	VN
56	Fusiform gyrus	FFG.R	VN
57	Postcentral gyrus	PoCG.L	MAN
58	Postcentral gyrus	PoCG.R	MAN
59	Superior parietal gyrus	SPG.L	MAN
60	Superior parietal gyrus	SPG.R	MAN
61	Inferior parietal, but supramarginal and angular gyri	IPL.L	AN
62	Inferior parietal, but supramarginal and angular gyri	IPL.R	AN
63	Supramarginal gyrus	SMG.L	MAN
64	Supramarginal gyrus	SMG.R	MAN
65	Angular gyrus	ANG.L	AN
66	Angular gyrus	ANG.R	AN
67	Precuneus	PCUN.L	DMN
68	Precuneus	PCUN.R	DMN
69	Paracentral lobule	PCL.L	MAN
70	Paracentral lobule	PCL.R	MAN
71	Caudate nucleus	CAU.L	LSN
72	Caudate nucleus	CAU.R	LSN
73	Lenticular nucleus, putamen	PUT.L	LSN
74	Lenticular nucleus, putamen	PUT.R	LSN

75	Lenticular nucleus, pallidum	PAL.L	LSN
76	Lenticular nucleus, pallidum	PAL.R	LSN
77	Thalamus	THA.L	LSN
78	Thalamus	THA.R	LSN
79	Heschl gyrus	HES.L	MAN
80	Heschl gyrus	HES.R	MAN
81	Superior temporal gyrus	STG.L	MAN
82	Superior temporal gyrus	STG.R	MAN
83	Temporal pole: superior temporal gyrus	TPOsup.L	AN
84	Temporal pole: superior temporal gyrus	TPOsup.R	MAN
85	Middle temporal gyrus	MTG.L	DMN
86	Middle temporal gyrus	MTG.R	DMN
87	Temporal pole: middle temporal gyrus	TPOmid.L	LSN
88	Temporal pole: middle temporal gyrus	TPOmid.R	LSN
89	Inferior temporal gyrus	ITG.L	AN
90	Inferior temporal gyrus	ITG.R	DMN

Table S2. P-values before and after correction of dynamic reconfiguration metrics at the subnetwork level

**Metrics**	**p-value**					**p-value (FDR)**				
	**MAN**	**VN**	**AN**	**DMN**	**LSN**	**MAN**	**VN**	**AN**	**DMN**	**LSN**
Inter-layer coupling	-	-	-	-	0.002	-	-	-	-	0.008
Promiscuity	0.030	-	-	-	-	0.049	-	-	-	-
Recruitment	0.026	-	0.008	0.014	-	0.043	-	0.038	0.034	-
Integration	-	-	-	-	0.003	-	-	-	-	0.014

Table S3. P-values before and after correction of inter-layer coupling at the node level

**ROI**	**Name**	**Abbreviation**	**Subnetwork**	**P**	**P(FDR)**
84	Temporal_Pole_Sup_R	TPOsup. R	MAN	0.0003	0.027
88	Temporal_Pole_Mid_R	TPOmid. R	LSN	0.001	0.037

Table S4. P-values before and after correction of recruitment at the node level

ROI	Name	Abbreviation	Subnetwork	P	P(FDR)
7	Frontal_Mid_L	MFG.L	AN	0.003	0.046
8	Frontal_Mid_R	MFG.R	AN	0.001	0.033
16	Frontal_Inf_Orb_R	ORBinf.R	AN	0.004	0.047
21	Olfactory_L	OLF.L	LSN	0.002	0.038
55	Fusiform_L	FFG.L	VN	0.0003	0.028

Table S5. P-values before and after correction of integration at the node level

ROI	Name	Abbreviation	Subnetwork	P	P(FDR)
27	Rectus_L	REC.L	DMN	0.001	0.046
37	Hippocampus_L	HIP.L	LSN	0.002	0.043
40	ParaHippocampal_R	PHG.R	LSN	0.004	0.04
41	Amygdala_L	AMYG.L	LSN	0.004	0.044
42	Amygdala_R	AMYG.R	LSN	0.003	0.038
75	Pallidum_L	PAL.L	LSN	0.001	0.033
76	Pallidum_R	PAL.R	LSN	0.005	0.045
87	Temporal_Pole_Mid_L	TPOmid.L	LSN	0.007	0.049
88	Temporal_Pole_Mid_R	TPOmid.R	LSN	0.001	0.039
89	Temporal_Inf_L	ITG.L	AN	0.003	0.040
90	Temporal_Inf_R	ITG.R	DMN	0.006	0.048
